# A Vitamin B_2_‐Photocatalysed Approach to Methionine Analogues

**DOI:** 10.1002/ange.202212158

**Published:** 2022-11-10

**Authors:** Oliver J. Knowles, Linus O. Johannissen, Giacomo E. M. Crisenza, Sam Hay, David Leys, David J. Procter

**Affiliations:** ^1^ Department of Chemistry University of Manchester Oxford Road Manchester M13 9PL UK; ^2^ Manchester Institute of Biotechnology and Department of Chemistry University of Manchester Princess Street Manchester M1 7DN UK

**Keywords:** methionine, flavins, photocatalysis, sulfides, amino acids

## Abstract

Access to new non‐canonical amino acid residues is crucial for medicinal chemistry and chemical biology. Analogues of the amino acid methionine have been far less explored—despite their use in biochemistry, pharmacology and peptide bioconjugation. This is largely due to limited synthetic access. Herein, we exploit a new disconnection to access non‐natural methionines through the development of a photochemical method for the radical α‐C−H functionalization of sulfides with alkenes, in water, using inexpensive and commercially‐available riboflavin (vitamin B_2_) as a photocatalyst. Our photochemical conditions allow the two‐step synthesis of novel methionine analogues—by radical addition to unsaturated amino acid derivatives—and the chemoselective modification of peptide side‐chains to yield non‐natural methionine residues within small peptides. The mechanism of the bio‐inspired flavin photocatalysis has been probed by experimental, DFT and TDDFT studies.

## Graphical abstract

Despite their emerging role in medicinal and biological chemistry, synthetic methods to access non‐natural methionine analogues remain underdeveloped. Using vitamin B_2_‐photocatalysis, simple sulfides undergo radical α‐C−H functionalization with unsaturated amino acid partners to deliver an array of new methionine derivatives. The approach also facilitates the chemoselective modification of peptide side‐chains.

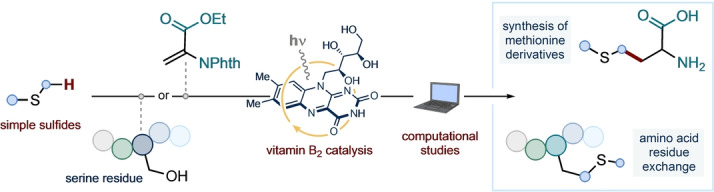

## Introduction

The incorporation of non‐canonical amino acids (ncAAs) into peptides and proteins has expanded the “genetic lexicon” of Nature beyond the twenty proteinogenic amino acids, and their post‐translational modification.^[1]^ Genetically encoded ncAA residues provide engineered peptides with bespoke structural, biological and pharmacological properties. In biocatalysis, the site‐selective incorporation of ncAAs enables the design of enzymes with enhanced efficiency or novel, non‐canonical catalytic functions.^[2]^ In medicinal chemistry and chemical biology, ncAA residues can be incorporated to fine‐tune the conformation, permeability, metabolic stability and potency of prominent peptide based therapeutics—including macrocyclic and linear oligopeptides, peptidomimetics, enzymes and antibodies.^[3]^ The increasing relevance of these biogenetic strategies has led the synthetic community to develop expedient, selective strategies to access broad arrays of new ncAAs.^[4]^

**Scheme 1:**
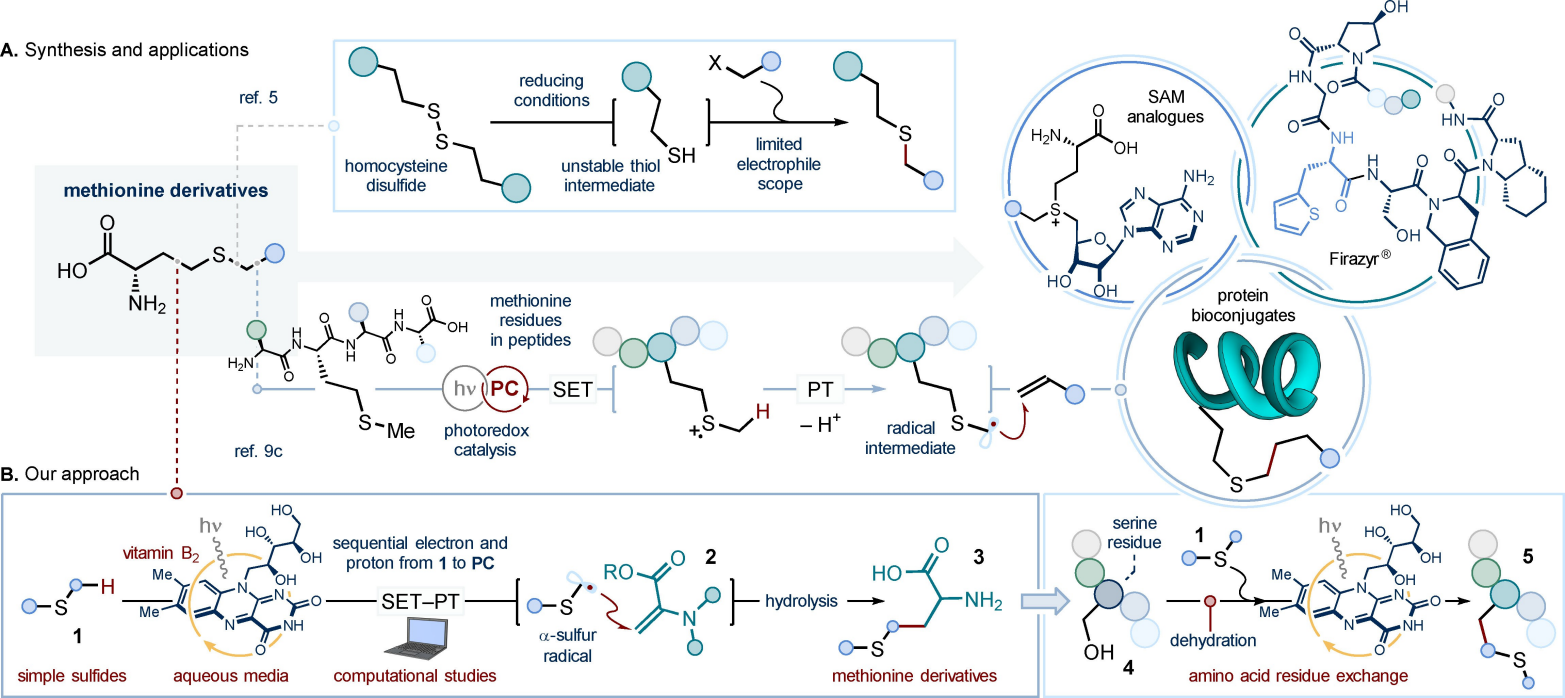
A. Synthetic routes to non‐natural methionine analogues, and the relevance of such analogues in chemical biology, medicinal chemistry and peptide bioconjugation. B. The development of a vitamin B_2_‐photocatalyzed radical coupling between simple sulfides and unsaturated amino acids streamlines the synthesis of methionine analogues (*our approach*), and their installation within small peptides (*residue exchange strategy*). PC: photocatalyst; PT: proton transfer; SAM: *S*‐adenosyl methionine; SET: single electron transfer. The series of colored spheres represents a peptide chain. Dashed lines denote retrosynthetic disconnections.

Against this backdrop, derivatives of the amino acid methionine have so far received less attention. This is perhaps due to the low abundance of this residue in vertebrates, and the lack of general and mild methods for their synthesis.^[5]^ Current synthetic approaches to methionine analogues rely on the in situ formation of oxygen‐sensitive homocysteine thiol intermediates—from the corresponding disulfide precursors under forcing, strictly anaerobic reduction conditions—which can undergo *S*‐alkylation with a limited selection of activated electrophilic partners (Scheme 1A, top).^[5]^ Crucially, however, methionine residues perform important functions in peptides—encompassing redox regulation,^[6]^ protection against oxidative stress,[^6a^, ^7^] and metal‐binding[^6d^, ^8^]—and its derivatives are key components of enzyme co‐factors (in particular, *S*‐adenosylmethionine and its analogues),[^5a^, ^5b^, ^5c^, ^5d^] marketed drugs (e.g. Firazyr®; a remedy for hereditary angioedema), and naturally‐occurring bioactive compounds (e.g. the albomycin and ezomycin classes of antibiotic).

The propensity of the sulfide functionality for oxidative modifications, combined with methionine's low abundance, make this residue an ideal target for both signaling and bioconjugation endeavors.^[9]^ Recent contributions by Toste and Chang,^[9a]^ Gaunt,^[9b]^ and MacMillan^[9c]^ have exploited the unique redox responsiveness of methionine's sulfur atom to design synthetic procedures for the selective functionalization of methionine residues in proteins and antibodies. In contrast with the first two bioconjugation methods[^9a^, ^9b^]—where the functionalization occurs at the *S*‐center of the amino acid residue—the photoredox strategy developed by MacMillan enables functionalization selectively at the α‐positions of the sulfide moiety of methionine (Scheme 1A, bottom).^[9c]^ Here, an excited‐state lumiflavin photocatalyst^[10]^ was used to promote the single electron transfer (SET) oxidation of the sulfur atom of methionine residues, forming the corresponding radical cation. Ensuing base‐assisted deprotonation at the adjacent sites (PT, proton transfer) generates α‐sulfur‐radicals, which can be intercepted by alkene radical traps,^[11]^ to provide the protein bioconjugates.

Inspired by this approach, we envisaged a new disconnection to access methionine derivatives (red dotted line in Scheme 1A); simple, readily available and unactivated sulfides **1** could be used as precursors for α‐sulfur‐radical intermediates—formed under photoredox conditions—that engage unsaturated amino acid traps **2** in a Giese type addition.^[4h]^ Such a process would deliver—upon hydrolysis—diversely functionalized methionine derivatives **3** (Scheme 1B). Herein, we demonstrate that the use of inexpensive and commercially‐available vitamin B_2_ (riboflavin) as the photocatalyst^[10f]^—under blue light irradiation—enables the α‐selective C−H functionalization of sulfides with traps of the type **2**—as well as a broad array of electron‐deficient alkene partners—thus allowing the two‐step synthesis of novel methionine analogues. Crucially, the use of vitamin B_2_ bypasses the need for an external base for proton transfer and radical generation, and also facilitates the use of aqueous media for the photochemical process—this being well suited to the manipulation of peptides. The latter aspect prompted us to apply our photochemical approach to the chemoselective modification of peptide side‐chains;^[12]^ serine residues were converted into both natural and non‐canonical methionine side‐chains within small polypeptides (*residue exchange strategy*, from **4** to **5**). Furthermore, computational studies have been used to shed light on the mechanistic steps underlying the bio‐inspired flavin photocatalysis.

## Results and Discussion

At the outset of our investigation, we sought to identify user‐friendly reaction conditions to promote the α‐selective C−H functionalization of sulfides in water, under vitamin B_2_‐photocatalysis. The reaction between tetrahydrothiophene **1 a** and phenyl vinyl sulfone **6 a**, delivering addition product **7 a**, was selected as a benchmark process (Table 1).^[13]^ Pleasingly, when a 20 mM solution of **6 a** in water was exposed to blue‐light irradiation (λ_max_ centered at 456 nm), at ambient temperature, and in the presence of 3 equivalents of **1 a** and 10 mol % of vitamin B_2_, product **7 a** was obtained in 12 % yield (entry 3). Conversely, when the reaction was conducted in organic solvents—such as DMF or methanol (entries 1–2)—**7 a** was only observed in trace amounts. Decreasing the concentration of the reaction to 5 mM (entry 4), and adding 5 % v/v of DMF (entry 5)—to facilitate the solubility of the organic reagents in the aqueous media—proved to be beneficial to the photochemical protocol. Ultimately, the use of larger amounts of inexpensive **1 a** (10 equiv.) produced **7 a** in 76 % isolated yield (entry 7). Crucially, the use of both flavin catalyst and light irradiation are essential (entries 8–9), whilst the use of much higher‐priced lumiflavin as the photocatalyst yielded **7 a** with reduced efficiency (entry 6).

**Table 1:** Process optimization.^[a]^

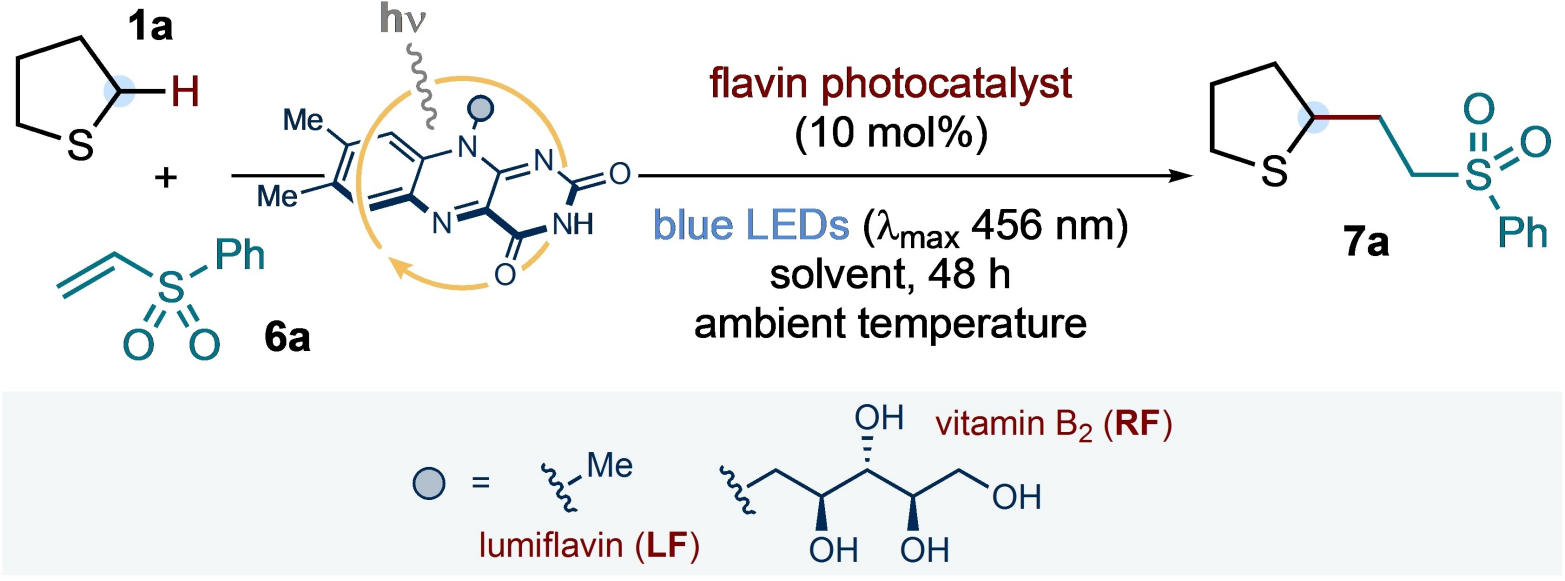				
entry	flavin PC	solvent (*c* mM)	equiv. **1 a**	yield **7 a**
1	**RF**	DMF (20 mM)	3	<5%
2	**RF**	MeOH (20 mM)	3	<5%
3	**RF**	H_2_O (20 mM)	3	12 %
4	**RF**	H_2_O (5 mM)	3	45 %
5	**RF**	5 % DMF:H_2_O (5 mM)	3	60 %
6	**LF**	5 % DMF:H_2_O (5 mM)	3	50 %
7	**RF**	5 % DMF:H_2_O (5 mM)	10	(76 %)
8	–	5 % DMF:H_2_O (5 mM)	10	NR
9^a^	**RF**	5 % DMF:H_2_O (5 mM)	10	NR

Reaction conditions: **1 a** (3–10 equiv.), **6 a** (50.0 μmol, 1 equiv.), flavin photocatalyst (10 mol %), in the appropriate solvent system at room temperature (∼25 °C, as controlled by the use of a fan) for 48 h, under irradiation by blue LEDs (λ_max_ centered at 456 nm, maximum irradiance). Yields were determined by ^1^H NMR spectroscopy using MeNO_2_ as internal standard. Isolated yields in parentheses. [a] Reaction performed in the dark. LED: light‐emitting diode; LF: lumiflavin; NR: no reaction, PC: photocatalyst; RF: riboflavin/vitamin B_2_.

**Scheme 2:**
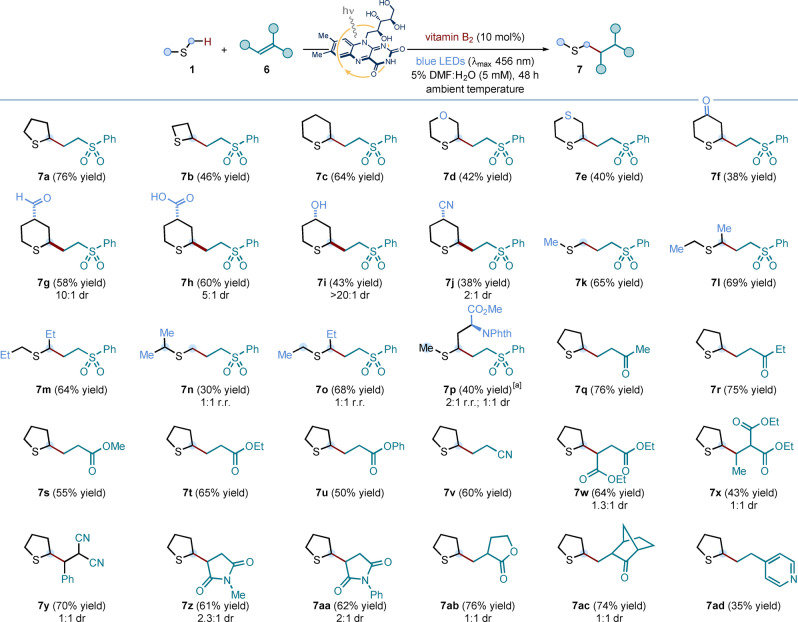
Scope of the vitamin B_2_‐photocatalyzed α‐C−H functionalization of sulfides with electron‐deficient alkene radical traps. Reaction conditions: **6** (0.1 mmol, 1 equiv.), **1** (10 equiv.), vitamin B_2_ (10 mol %), in 5 % v/v DMF:H_2_O (5 mM with respect to **6**) at room temperature (∼25 °C, as controlled by the use of a fan), under irradiation by blue LEDs (λ_max_ centered at 456 nm, maximum irradiance) for 48 h. Isolated yields are given in parentheses. The reported diastereoselectivity was determined by ^1^H NMR analysis of the purified compounds.^[a]^ Reaction run in the presence of 3 equiv. of sulfide **1 p**. NPhth: *N*‐Phthalimide.

With efficient conditions for the vitamin B_2_‐photocatalyzed α‐C−H functionalization of sulfides in hand, we sought to explore the scope of the transformation with respect to both the sulfide partner and the alkene radical trap (Scheme 2). Besides tetrahydrothiophene **1 a**, alternative cyclic sulfides—including thietane, thiane, thioxane, dithiane, and thianone—participated in the light‐driven reactivity with **6 a**, giving the corresponding addition products **7 b**–**f** in good to moderate yield. The thiane core was selected to evaluate the functional group tolerance of our photochemical conditions: aldehyde **7 g**, carboxylic acid **7 h**, alcohol **7 i**, and nitrile **7 j** were all obtained in good yield and with diastereocontrol; alcohol **7 i** was obtained as a single diastereoisomer. (See the Supporting Information for a discussion of the possible origin of diastereocontrol). Linear sulfides were also competent substrates for the photochemical α‐C−H functionalization reaction with **6 a** (products **7 k**–**o**); although, when asymmetrically substituted, these led to the formation of equimolar mixtures of regioisomers (**7 n**–**o**). Interestingly, protected methionine **1 p** underwent C−H functionalization prevalently at the α‐methylene site (**7 p** obtained in 2 : 1 r.r. and 1 : 1 dr). Exploring the scope with respect to the radical trap, a range of electron‐deficient alkene substrates—including vinyl ketones, acrylates, acrylonitrile, ethyl fumarate, ethyl ethylidenemalonate, benzylidenemalononitrile, and maleimides—were subjected to the vitamin B_2_‐photocatalyzed protocol; using tetrahydrothiophene **1 a**, addition products **7 q**–**aa** were obtained in good yields. In addition, an unsaturated lactone, a norbornane derivative, and 4‐vinylpyridine could also be successfully coupled with **1 a** to give products **7 ab**, **7 ac** and **7 ad**, respectively.

**Scheme 3:**
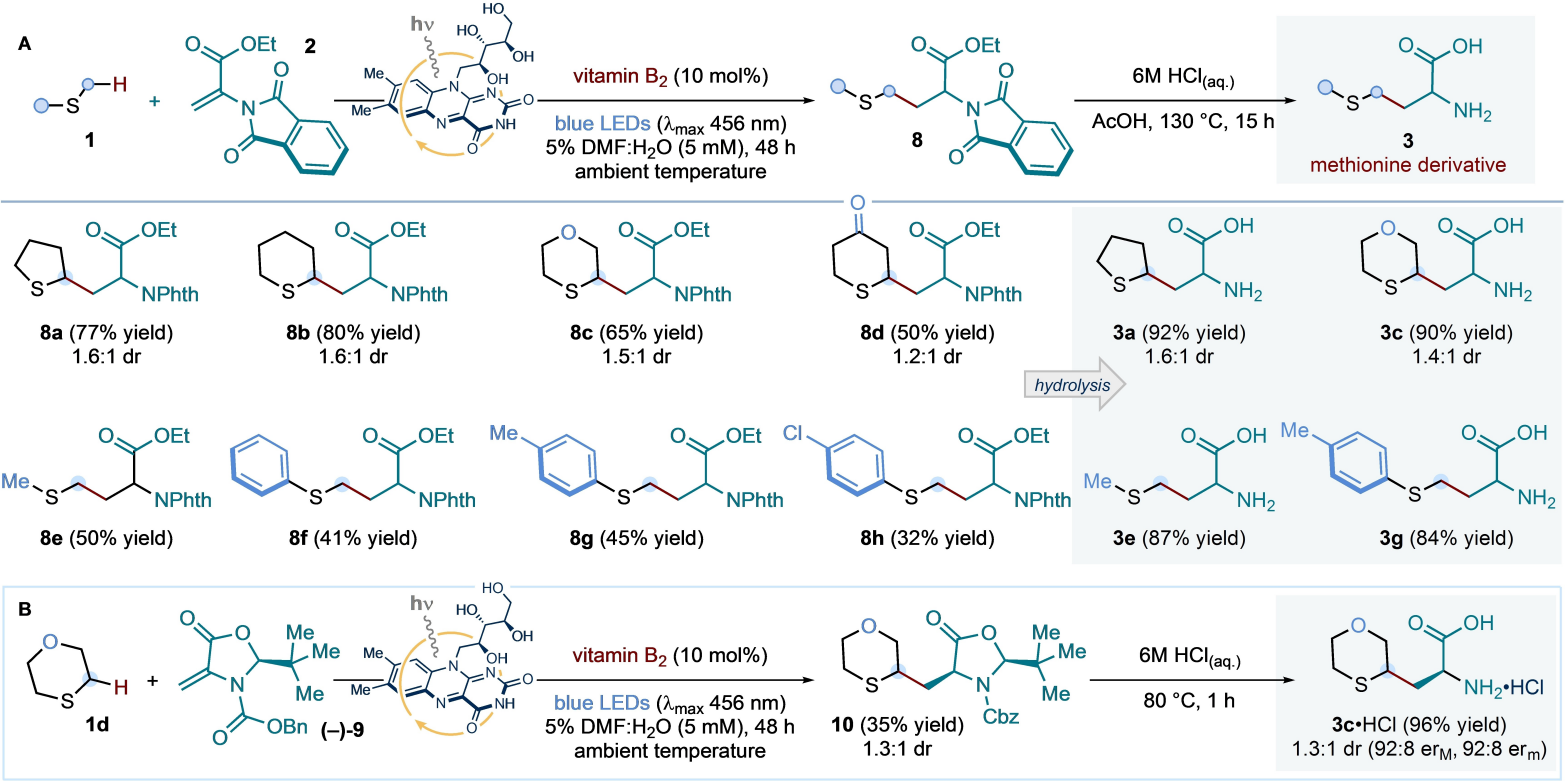
A. Application of the vitamin B_2_‐photocatalyzed process to the synthesis of methionine analogues **3**. Conditions: **2** (0.1 mmol, 1 equiv.), **1** (10 equiv.), vitamin B_2_ (10 mol %), in 5 % v/v DMF:H_2_O (5 mM with respect to **2**) at room temperature (∼25 °C, as controlled by the use of a fan), under irradiation by blue LEDs (λ_max_ centered at 456 nm, maximum irradiance) for 48 h; followed by treatment of product **8** with aq. 6 M HCl in AcOH, at 130 °C, for 15 h. B. Preliminary investigations towards the development of an asymmetric variant for the protocol. Conditions: **(−)‐9** (0.1 mmol, 1 equiv.), **1 d** (10 equiv.), vitamin B_2_ (10 mol %), in 5 % v/v DMF:H_2_O (5 mM with respect to **(−)‐9**) at room temperature (∼25 °C, as controlled by the use of a fan), under irradiation by blue LEDs (λ_max_ centered at 456 nm, maximum irradiance) for 48 h; followed by treatment of **10** with aq. 6 M HCl, at 80 °C, for 1 h. Isolated yields are given in parentheses. The reported diastereoselectivity was determined by ^1^H NMR analysis of the purified compounds; while ee values were measured by HPLC analyses on a reverse chiral stationary phase. NPhth: *N*‐Phthalimide.

We next sought to apply our expedient, mild and robust photocatalytic protocol to the synthesis of methionine analogues by means of a two‐step radical addition/hydrolysis sequence (Scheme 3A). A selection of cyclic sulfides—specifically, tetrahydrothiophene, thiane, thioxane, and thianone—were exposed to the optimized photoredox conditions in the presence of unsaturated amino acid **2**. These experiments delivered adducts **8 a**–**d** in good to excellent yield, and with moderate diastereoselectivity. Dimethyl sulfide and three thioanisole derivatives also proved to be competent radical precursors for the vitamin B_2_‐photocatalyzed addition to alkene **2**; yielding protected methionine **8 e** and its *S*‐aryl analogues **8 f**–**h**, respectively. Crucially, the latter compounds would be hard‐to‐attain using direct *S*‐arylation methods^[13]^—involving homocysteine thiol intermediates—thus showcasing the synthetic advantage brought about by the new disconnection.

Adducts **8 a**, **8 c**, **8 e** and **8 g** were used to develop efficient hydrolysis conditions: in all cases, heating a solution of **8** in 6 M HCl and acetic acid cleanly delivered the corresponding unprotected amino acid derivatives **3** in excellent yields (Scheme 3A). Using the stereodefined Beckwith‐Karady alkene[^4h^, ^14^, ^15^] **(−)‐9** as the radical trap in the photochemical α‐C−H functionalization of thioxane **1 d**, under the previously optimized conditions, a diastereoselective addition of the α‐sulfur‐radical from **1 d** was achieved in 35 % yield, with control of the construction of the α‐stereocenter in the protected amino acid **10** (i.e. only two diastereoisomers were detected) (Scheme 3B). Finally, hydrolysis of adduct **10** with aqueous 6 M HCl delivered quantitatively a 1.3 : 1 diastereomeric mixture of enantioenriched cyclic methionine analogue **3 c**—isolated as the corresponding hydrochloride salt—thus providing an asymmetric variant of our photochemical route to methionine derivatives.

**Scheme 4:**
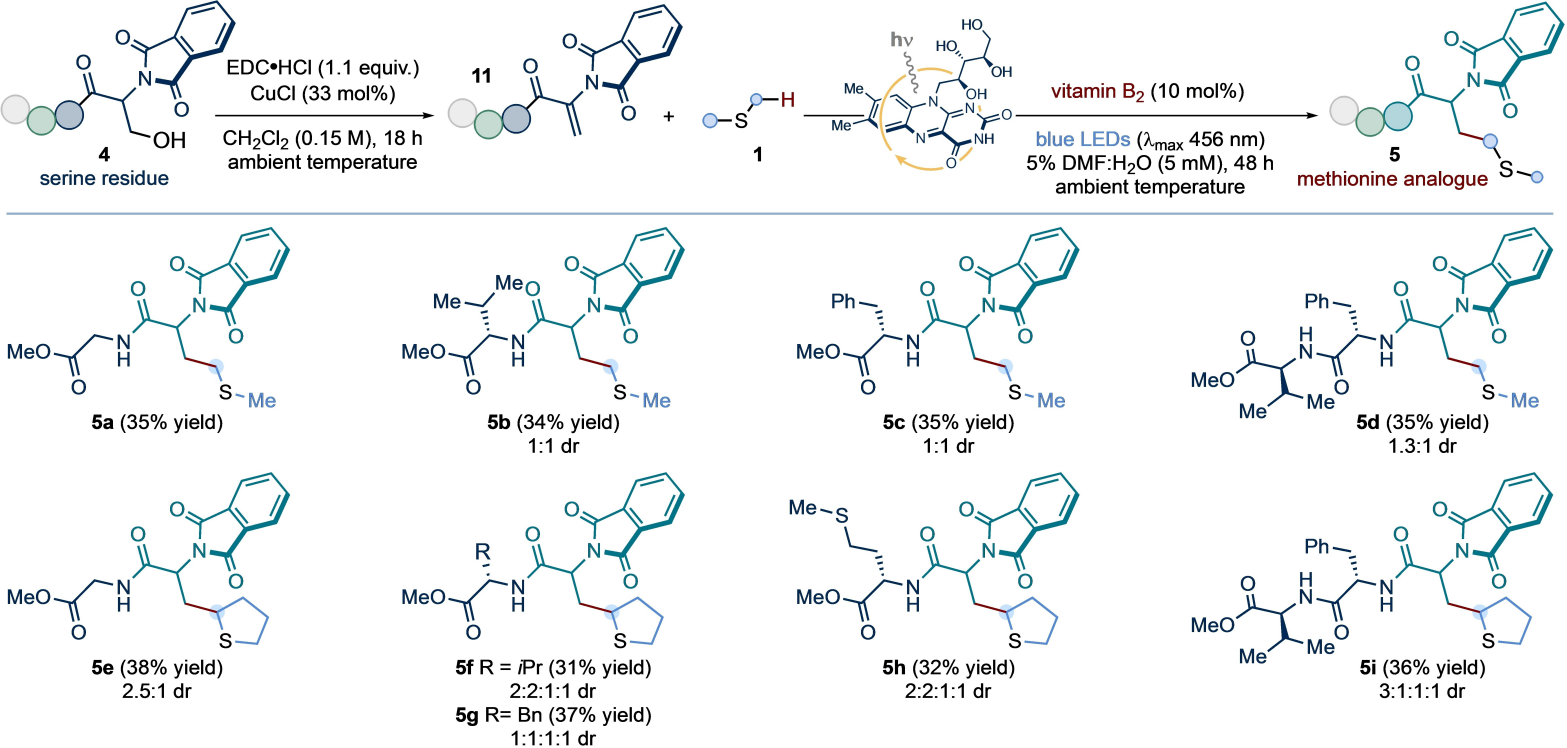
Application of the developed vitamin B_2_‐photocatalyzed conditions to the direct installation of both canonical and non‐natural methionine residues within small peptides, through a two‐step dehydration/radical addition sequence from serine side‐chains: *residue exchange strategy*. Conditions: **4** (1 equiv.), EDC ⋅ HCl (1.1 equiv.), CuCl (33 mol %), in CH_2_Cl_2_ (0.15 M with respect to **4**) at ambient temperature for 18 h; followed by exposure of product **11** (0.1 mmol, 1 equiv.) to **1** (10 equiv.), vitamin B_2_ (10 mol %), in 5 % v/v DMF:H_2_O (5 mM with respect to **11**) at room temperature (∼25 °C, as enabled by the use of a fan), under irradiation by blue LEDs (λ_max_ centered at 456 nm, maximum irradiance) for 48 h. Isolated yields are given in parentheses. The reported diastereoselectivity was determined by ^1^H NMR analysis of the purified compounds. NPhth: *N*‐Phthalimide.

We next investigated whether unsaturated residues (related to **2**) in small peptide structures could participate in the light‐driven coupling to construct, in situ, natural and unnatural methionine residues. Crucially, unsaturated amino acid residues can be expediently obtained through the Cu‐catalyzed dehydration of serine side‐chains.^[16]^ Thus, we designed a chemoselective amino acid modification protocol, whereby serine side‐chains are converted to methionine residues—or their analogues—via a two‐step sequence (*residue exchange strategy*, Scheme 4).

To test this strategy, terminal unsaturated di‐ and tri‐peptides **11**—obtained by treatment of the corresponding serine precursors **4** with *N*‐(3‐dimethylamino‐propyl)‐*N′*‐ethylcarbodiimide hydro‐chloride (EDC ⋅ HCl) and copper chloride—were submitted to the photocatalytic protocol, in water, in the presence of either dimethyl sulfide or tetrahydrothiophene. Under the previously optimized catalytic conditions, methionine derivatives **5 a**–**d** and tetrahydrothiophene analogues **5 e**–**i** were isolated in moderate yields. Crucially, a methionine‐containing unsaturated dipeptide underwent selective coupling with tetrahydrothiophene to give **5 h** with no intramolecular or intermolecular homocoupling observed. Our preliminary findings show that our photochemical approach may offer a facile, chemoselective platform for the direct modification of amino acid residues and the post‐translational modification of polypeptides.

**Scheme 5:**
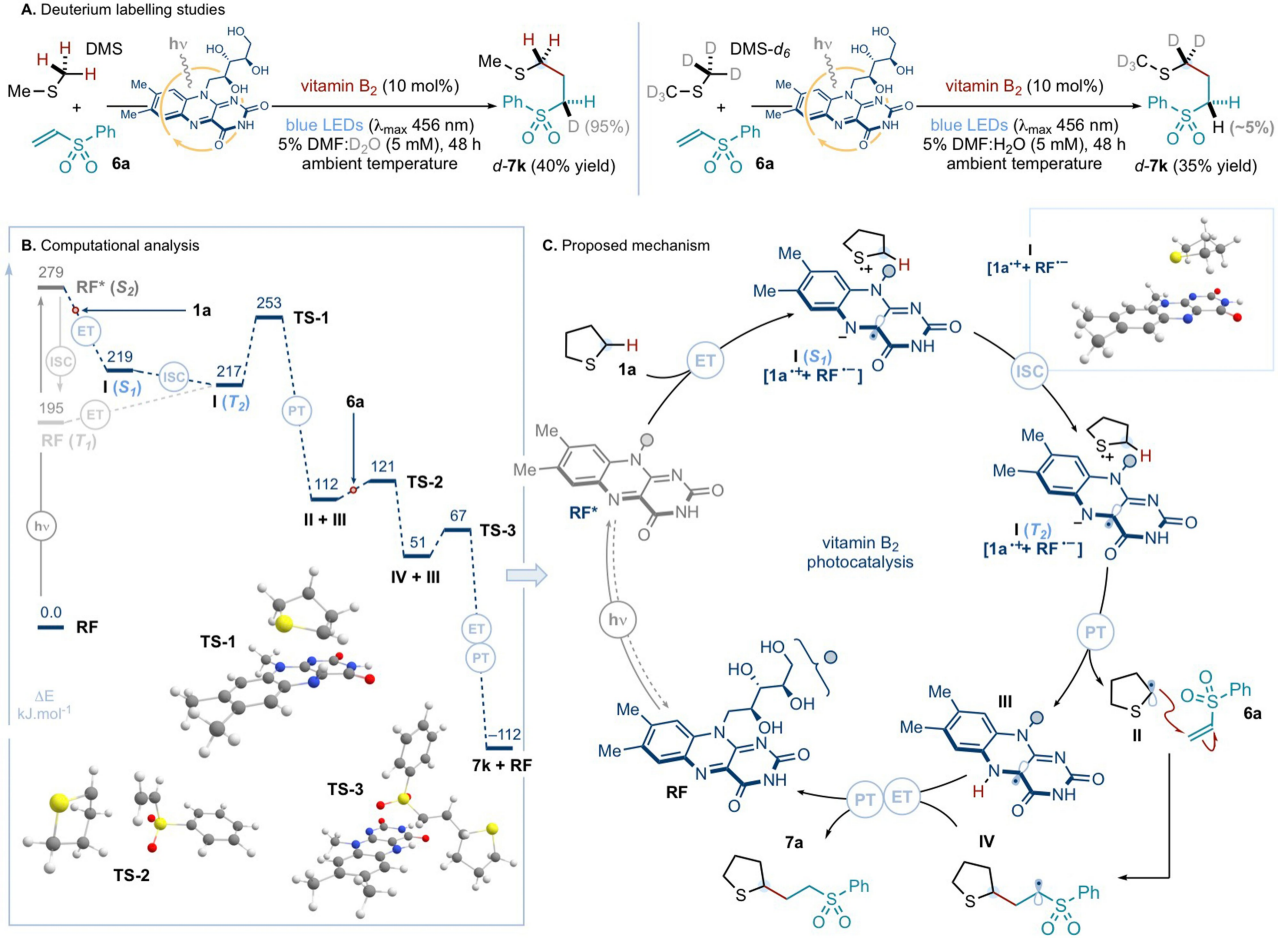
Mechanistic investigations: *A*. Deuterium labelling experiments. *B*. Energy profile for the photochemical reaction between **1 a** and **6 a**, catalyzed by **RF**, obtained via DFT and TDDFT calculations (ΔE values reported in kJ mol^−1^, using the B3LYP functional). The red circles on the dotted lines represents the addition of the given reagent (i.e. **1 a** and, then, **6 a**). *C*. Proposed catalytic cycle for the vitamin B_2_‐photocatalyzed coupling of **1 a** and **6 a**. ET: electron transfer; ISC: intersystem crossing; PT: proton transfer; TS: transition state.

After exploring its synthetic applications, the mechanism of the vitamin B_2_‐photocatalyzed α‐C−H coupling of sulfides with alkenes was examined. First, deuterium labelling experiments were undertaken to interrogate the nature of the final proton transfer event that delivers product (Scheme 5A). When the standard photochemical coupling between dimethyl sulfide (**DMS**) and vinyl sulfone **6 a** was conducted in a 5 % mixture of DMF in deuterium oxide (D_2_O), quantitative deuterium incorporation at the methylene site adjacent to the sulfone moiety was observed. This was not the case when the experiment was performed in a 5 % DMF:H_2_O solution, using deuterated **DMS**‐*d*_6_ and **6 a** as substrates: here, under the previously employed photochemical conditions, a negligible amount (∼5%) of deuterium incorporation at the methylene site α to the sulfone was detected. This study suggests that the dominant reaction pathway involves reduction of the radical intermediate—generated after the addition of the α‐sulfur radical to the alkene trap—by the reduced form of the flavin photocatalyst, and protonation of the resulting anionic intermediate (not shown) by the aqueous media (see below). Nevertheless, an alternative scenario in which proton scrambling between the media and the protonated and reduced form of the flavin catalyst occurs, followed by hydrogen atom transfer (HAT) to the carbon‐centered radical, cannot be ruled out.

To gain further understanding of the mechanistic steps underpinning the vitamin B_2_‐photocatalysis, computational modelling—using DFT and time‐dependent DFT (TDDFT) calculations—was deployed (computational methods are detailed in the Supporting Information). The computed energy profile for the photochemical reaction between tetrahydrothiophene **1 a** and phenyl vinyl sulfone **6 a**, catalyzed by riboflavin (**RF**, vitamin B_2_), was examined using both the B3LYP and M06‐2X functionals (only B3LYP shown in Scheme 5B; M06‐2X results are qualitatively identical, as shown in the Supporting Information). According to our TDDFT calculations, electron transfer does not occur from **1 a** to **RF** in the ground‐state, but requires the photoexcitation of **RF**, under blue‐light irradiation, with an energetic cost of 279 kJ mol^−1^ (429 nm). After visible‐light excitation, exergonic photoinduced SET from **1 a** to RF* forms radical ion pair **I** [**1 a**⋅^+^+**RF**⋅^−^] in its singlet‐*S*_1_ state (Supporting Information Figure S10). The calculated barrier for the ensuing proton transfer (PT) event from **1 a**⋅^+^ to **RF**⋅^−^, within **I** in its singlet state, would be energetically prohibitive (ΔE>100 kJ mol^−1^ calculated using both functionals; see Supporting Information Figure S11); while the PT process has a low, exothermic barrier when complex **I** is in its triplet state (see below). This analysis implies that **I** undergoes intersystem crossing (ISC) to its triplet state prior to the PT event. Crucially, the calculated spin‐orbit couplings (SOC, computed using PySOC^13^, see Supporting Information) for **I** suggest that the formation of the radical ion pair greatly enhances the probability of intersystem crossing (ISC). Accordingly, the computations suggest that the barrier for the ISC [**I (*S***_**1**_ **)**→**I (*T***_**2**_ **)**] is low and exergonic (ΔE −2 kJ mol^−1^). In contrast, the alternative scenario in which triplet complex **I (*T***_**2**_ **)** is formed upon ISC of singlet‐state photoexcited RF* (*S*_**2**_ **)** to RF* (*T*_**1**_ **)**, followed by ET to sulfide **1 a** (grey pathway in Scheme 5B), is less energetically favorable (ΔE for ET +22 kJ mol^−1^) in our models. Our DFT calculations then suggest that highly exergonic intracomplex PT in **I (*T***_**2**_ **)**—from **1 a**⋅^+^ to **RF**⋅^−^ (ΔE −105 kJ mol^−1^)—generates, via transition state **TS‐1**, open‐shell intermediate **II** along with the reduced and protonated form of the photocatalyst (**III**). At this stage, facile radical addition of **II** to alkene trap **6 a** (ΔE −61 kJ mol^−1^) produces radical intermediate **IV**, via **TS‐2**. The latter species is rapidly reduced and protonated by **III** (ΔE −163 kJ mol^−1^), through **TS‐3**, to deliver the α‐C−H functionalized sulfide and restore ground‐state **RF**.

According to the insights provided by both the experimental and computational studies, we propose a plausible catalytic cycle for the light‐driven α‐C−H coupling of tetrahydrothiophene **1 a** and **6a**, mediated by vitamin B_2_ (Scheme 5C). Photoexcitation of the ground‐state **RF**, under blue‐light irradiation, produces excited‐state RF*. This is reduced by SET from the sulfide **1 a**, resulting in the formation of radical ion pair **I** [**1 a**⋅^+^+**RF**⋅^−^] in its singlet *S_1_*‐state (See Supporting Information for fluorescence quenching studies and UV/Vis studies; the latter suggesting that pre‐association of flavin and sulfide does not occur). Complex **I** undergoes ISC to its triplet *T_2_*‐state in order to enable the proton transfer event from **1 a**⋅^+^ to **RF**⋅^−^.^17^ The latter step generates α‐sulfur radical **II**, together with the protonated and reduced form of the riboflavin catalyst **III**. Addition of **II** to electron‐deficient alkene trap **6a** delivers open‐shell intermediate **IV**, which—as suggested by the studies in Scheme 5A—is first reduced by SET from **III** and, then, protonated by the aqueous media to deliver protected methionine derivative **7a** and restoring the ground‐state photocatalyst **RF**.

## Conclusion

An expedient, mild and selective radical α‐C−H coupling of sulfides and alkenes allows synthetic access to diverse methionine derivatives. The photochemical protocol proceeds in water, uses simple and readily available starting materials, and is facilitated by the photocatalytic activity of inexpensive vitamin B_2_ (riboflavin). In addition to providing a new disconnection en route to sulfur‐containing amino acids, our approach has been rendered stereoselective, through the use of a stereodefined radical trap, and has been applied in the direct modification of amino acid residues in small polypeptides; this residue exchange strategy allows methionine residues, and their analogues, to be installed at the expense of serine residues. Finally, time‐dependent computational analysis has shed light on the mechanistic steps underpinning the light‐driven vitamin B_2_‐catalysis. These computational studies indicate that the formation of a radical ion pair between the oxidized sulfide and the reduced riboflavin, and ensuing ISC from its *S_1_*‐ to *T_2_*‐state, is crucial for formation of the α‐sulfur radical and the observed photochemical reactivity.

## Conflict of interest

The authors declare no conflict of interest.

1

## Supporting information

As a service to our authors and readers, this journal provides supporting information supplied by the authors. Such materials are peer reviewed and may be re‐organized for online delivery, but are not copy‐edited or typeset. Technical support issues arising from supporting information (other than missing files) should be addressed to the authors.

Supporting Information

## Data Availability

The data that support the findings of this study are available in the supplementary material of this article.

## References

[1] For discussion and perspectives, see:

[1a] T. S. Young , P. G. Schultz , J. Biol. Chem. 2010, 285, 11039–11044; 20147747 10.1074/jbc.R109.091306 PMC2856976

[1b] I. Drienovská , G. Roelfes , Nat. Catal. 2020, 3, 193–202;

[1c] J. B. Hedges , K. S. Ryan , Chem. Rev. 2020, 120, 3161–3209. 31869221 10.1021/acs.chemrev.9b00408

[2] Selected reviews:

[2a] J. A. Johnson , Y. Y. Lu , J. A. Van Deventer , D. A. Tirrell , Curr. Opin. Struct. Biol. 2010, 14, 774–780; 10.1016/j.cbpa.2010.09.013 PMC3008400 21071259

[2b] S. Zheng , I. Kwon , Biotechnol. J. 2012, 7, 47–60; 22121038 10.1002/biot.201100267

[2c] P. Neumann-Staubitz , H. Neumann , Curr. Opin. Struct. Biol. 2016, 38, 119–128; 27318816 10.1016/j.sbi.2016.06.006

[2d] J. Zhao , A. J. Burke , A. P. Green , Curr. Opin. Struct. Biol. 2020, 55, 136–144; 10.1016/j.cbpa.2020.01.006 32163871

[2e] S. L. Lovelock , R. Crawshaw , S. Basler , C. Levy , D. Baker , D. Hilvert , A. P. Green , Nature 2022, 606, 49–58. 35650353 10.1038/s41586-022-04456-z

[3] For discussion, see:

[3a] M. A. T. Blaskovich , J. Med. Chem. 2016, 59, 10807–10836; 27589349 10.1021/acs.jmedchem.6b00319

[3b] M. Erak , K. Bellmann-Sickert , S. Els-Heindl , A. G. Beck-Sickinger , Bioorg. Med. Chem. 2018, 26, 2759–2765; 29395804 10.1016/j.bmc.2018.01.012

[3c] A. Rezhdo , M. Islam , M. Huang , J. A. Van Deventer , Curr. Opin. Biotechnol. 2019, 60, 168–178; 30974337 10.1016/j.copbio.2019.02.020 PMC6783319

[3d] M. Muttenthaler , G. F. King , D. J. Adams , P. F. Alewood , Nat. Rev. Drug Discovery 2021, 20, 309–325. 33536635 10.1038/s41573-020-00135-8

[4] Selected recent contributions:

[4a] R. A. Aycock , D. B. Vogt , N. T. Jui , Chem. Sci. 2017, 8, 7998–8003; 29619169 10.1039/c7sc03612d PMC5863445

[4b] D. J. Leonard , J. W. Ward , J. Clayden , Nature 2018, 562, 105–109; 30283103 10.1038/s41586-018-0553-9

[4c] S. Ni , A. F. Garrido-Castro , R. R. Merchant , J. N. de Gruyter , D. C. Schmitt , J. J. Mousseau , G. M. Gallego , S. Yang , M. R. Collins , J. X. Qiao , K.-S. Yeung , D. R. Langley , M. A. Poss , P. M. Scola , T. Qin , P. S. Baran , Angew. Chem. Int. Ed. 2018, 57, 14560–14565; 10.1002/anie.201809310 PMC6352899 30212610

[4d] Z.-P. Yang , D. J. Freas , G. C. Fu , J. Am. Chem. Soc. 2021, 143, 8614–8618; 34080836 10.1021/jacs.1c03903 PMC8351905

[4e] T. M. Faraggi , C. Rouget-Virbel , J. A. Rincón , M. Barberis , C. Mateos , S. García-Cerrada , J. Agejas , O. de Frutos , D. W. C. MacMillan , Org. Process Res. Dev. 2021, 25, 1966–1973; 35663840 10.1021/acs.oprd.1c00208 PMC9162430

[4f] E. K. Edelstein , D. A. Rankic , C. C. Dudley , S. E. McMinn , D. A. Adpressa , ACS Catal. 2021, 11, 743–749;

[4g] C.-X. Ye , X. Shen , S. Chen , E. Meggers , Nat. Chem. 2022, 14, 566–573. For a recent review, see:35379900 10.1038/s41557-022-00895-3 PMC7612692

[4h] F. J. Aguilar Troyano , K. Merkens , K. Anwar , A. Gómez-Suárez , Angew. Chem. Int. Ed. 2021, 60, 1098–1115; 10.1002/anie.202010157 PMC7820943 32841470

[5] Selected protocols proceeding via the formation of homocysteine thiol intermediates:

[5a] M. Thomsen , S. B. Vogensen , J. Buchardt , M. D. Burkart , R. P. Clausen , Org. Biomol. Chem. 2013, 11, 7606–7610; 24100405 10.1039/c3ob41702f PMC3871182

[5b] S. Singh , J. Zhang , T. D. Huber , M. Sunkara , K. Hurley , R. D. Goff , G. Wang , W. Zhang , C. Liu , J. Rohr , S. G. Van Lanen , A. J. Morris , J. S. Thorson , Angew. Chem. Int. Ed. 2014, 53, 3965–3969; 10.1002/anie.201308272 PMC4076696 24616228

[5c] M. Dippe , W. Brandt , H. Rost , A. Porzel , J. Schmidt , L. A. Wessjohann , Chem. Commun. 2015, 51, 3637–3640; 10.1039/c4cc08478k 25642798

[5d] F. Muttach , A. Rentmeister , Angew. Chem. Int. Ed. 2016, 55, 1917–1920; 10.1002/anie.201507577 26694947

[5e] B. Bhushan , Y. A. Lin , M. Bak , A. Phanumartwiwath , N. Yang , M. K. Bilyard , T. Tanaka , K. L. Hudson , L. Lercher , M. Stegmann , S. Mohammed , B. G. Davis , J. Am. Chem. Soc. 2018, 140, 14599–14603; 30371070 10.1021/jacs.8b09433

[5f] A. K. Mishra , R. Tessier , D. P. Hari , J. Waser , Angew. Chem. Int. Ed. 2021, 60, 17963–17968. For an alternative disconnection using thiol substrates, see:10.1002/anie.202106458 PMC8456932 34038604

[5g] J. Jiracek , M. Collinsova , I. Rosenberg , M. Budesinsky , E. Protivinska , H. Netusilova , T. A. Garrow , J. Med. Chem. 2006, 49, 3982–3989; 16789755 10.1021/jm050885v PMC2622432

[5h] S. K. Patel , T. E. Long , Tetrahedron Lett. 2009, 50, 5067–5070.

[6a] R. L. Levine , J. Moskovitz , E. R. Stadtman , IUBMB Life 2000, 50, 301–307; 11327324 10.1080/713803735

[6b] R.-J. Hung , C. W. Pak , J. R. Terman , Science 2011, 334, 1710–1713; 22116028 10.1126/science.1211956 PMC3612955

[6c] B. C. Lee , Z. Péterfi , F. W. Hoffmann , R. E. Moore , A. Kaya , A. Avanesov , L. Tarrago , Y. Zhou , E. Weerapana , D. E. Fomenko , P. R. Hoffmann , V. N. Gladyshev , Mol. Cell 2013, 51, 397–404; 23911929 10.1016/j.molcel.2013.06.019 PMC4262529

[6d] A. Kaya , B. C. Lee , V. N. Gladyshev , Antioxid. Redox Signaling 2015, 23, 814–822; 10.1089/ars.2015.6385 PMC4589106 26181576

[6e] G. Kim , R. L. Levine , Biochemistry 2016, 55, 3586–3593. 27259041 10.1021/acs.biochem.6b00180 PMC5020372

[7a] R. L. Levine , L. Mosoni , B. S. Berlett , E. R. Stadtman , Proc. Natl. Acad. Sci. USA 1996, 93, 15036–15040; 8986759 10.1073/pnas.93.26.15036 PMC26351

[7b] J. Moskovitz , S. Bar-Noy , W. M. Williams , J. Requena , B. S. Berlett , E. R. Stadtman , Proc. Natl. Acad. Sci. USA 2001, 98, 12920–12925. 11606777 10.1073/pnas.231472998 PMC60800

[8] R. N. V. Krishna Deepak , B. Chandrakar , R. Sankararamakrishnan , Biophys. Chem. 2017, 224, 32–39. 28363089 10.1016/j.bpc.2017.02.007

[9a] S. Lin , X. Yang , S. Jia , A. M. Weeks , M. Hornsby , P. S. Lee , R. V. Nichiporuk , A. T. Iavarone , J. A. Wells , F. D. Toste , C. J. Chang , Science 2017, 355, 597–602; 28183972 10.1126/science.aal3316 PMC5827972

[9b] M. T. Taylor , J. E. Nelson , M. G. Suero , M. J. Gaunt , Nature 2018, 562, 563–568; 30323287 10.1038/s41586-018-0608-y PMC6203954

[9c] J. Kim , B. X. Li , R. Y.-C. Huang , J. X. Qiao , W. R. Ewing , D. W. C. MacMillan , J. Am. Chem. Soc. 2020, 142, 21260–21266; For a review, see:33290649 10.1021/jacs.0c09926 PMC8647115

[9d] J. Zang , Y. Chen , W. Zhu , S. Lin , Biochemistry 2020, 59, 132–138. 31592657 10.1021/acs.biochem.9b00789

[10] Selected recent synthetic applications of flavin-photocatalysis:

[10a] S. Bloom , C. Liu , D. K. Kölmel , J. X. Qiao , Y. Zhang , M. A. Poss , W. R. Ewing , D. W. C. MacMillan , Nat. Chem. 2018, 10, 205–211; 29359756 10.1038/nchem.2888 PMC6343675

[10b] T. Morack , J. B. Metternich , R. Gilmour , Org. Lett. 2018, 20, 1316–1319; 29446957 10.1021/acs.orglett.8b00052

[10c] M. Chilamari , J. R. Immel , S. Bloom , ACS Catal. 2020, 10, 12727–12737; 10.1021/acscatal.0c03422 PMC12622572 41257217

[10d] J. R. Immel , M. Chilamari , S. Bloom , Chem. Sci. 2021, 12, 10083–10091; 34377401 10.1039/d1sc02562g PMC8317666

[10e] R. Foja , A. Walter , C. Jandl , E. Thyrhaug , J. Hauer , G. Storch , J. Am. Chem. Soc. 2022, 144, 4721–4726; For a review, see:35259294 10.1021/jacs.1c13285

[10f] V. Srivastava , P. K. Singh , A. Srivastava , P. P. Singh , RSC Adv. 2021, 11, 14251–14259.

[11a] X.-Z. Fan , J.-W. Rong , H.-L. Wu , Q. Zhou , H.-P. Deng , J. D. Tan , C.-W. Xue , L.-Z. Wu , H.-R. Tao , J. Wu , Angew. Chem. Int. Ed. 2018, 57, 8514–8518; 10.1002/anie.201803220 29718584

[11b] E. Alfonzo , S. M. Hande , ACS Catal. 2020, 10, 12590–12595.

[12] For recent examples of direct peptide modification through radical reactivity, see:

[12a] A. Yang , S. Ha , J. Ahn , R. Kim , S. Kim , Y. Lee , J. Kim , D. Söll , H.-Y. Lee , H.-S. Park , Science 2016, 354, 623–626; 27708052 10.1126/science.aah4428 PMC5135561

[12b] T. H. Wright , B. J. Bower , J. M. Chalker , G. J. L. Bernardes , R. Wiewiora , W.-L. Ng , R. Raj , S. Faulkner , M. R. J. Vallée , A. Phanumartwiwath , O. D. Coleman , M.-L. Thézénas , M. Khan , S. R. G. Galan , L. Lercher , M. W. Schombs , S. Gerstberger , M. E. Palm-Espling , A. J. Baldwin , B. M. Kessler , T. D. W. Claridge , S. Mohammed , B. G. Davis , Science 2016, 354, aag1465; 27708059 10.1126/science.aag1465

[12c] B. Josephson , C. Fehl , P. G. Isenegger , S. Nadal , T. H. Wright , A. W. J. Poh , B. J. Bower , A. M. Giltrap , L. Chen , C. Batchelor-McAuley , G. Roper , O. Arisa , J. B. I. Sap , A. Kawamura , A. J. Baldwin , S. Mohammed , R. G. Compton , V. Gouverneur , B. G. Davis , Nature 2020, 585, 530–537; 32968259 10.1038/s41586-020-2733-7

[12d] R. C. Griffiths , F. R. Smith , J. E. Long , D. Scott , H. E. L. Williams , N. J. Oldham , R. Layfield , N. J. Mitchell , Angew. Chem. Int. Ed. 2022, 61, e202110223. 10.1002/anie.202110223 PMC9299887 34713958

[13] Selected examples:

[13a] A. Correa , M. Carril , C. Bolm , Angew. Chem. Int. Ed. 2008, 47, 2880–2883; 10.1002/anie.200705668 18318033

[13b] H.-J. Xu , Y.-Q. Zhao , T. Feng , Y.-S. Feng , J. Org. Chem. 2012, 77, 2878–2884; 22339180 10.1021/jo300100x

[13c] P. Yang , X. Wang , B. Li , Y. Yang , J. Yue , Y. Suo , H. Tong , G. He , X. Lu , G. Chen , Chem. Sci. 2021, 12, 5804–5810; For a review, see:34168804 10.1039/d1sc00789k PMC8179660

[13d] C. Zhang , E. V. Vinogradova , A. M. Spokoyny , S. L. Buchwald , B. L. Pentelute , Angew. Chem. Int. Ed. 2019, 58, 4810–4839; 10.1002/anie.201806009 PMC6433541 30399206

[14a] S. Karady , J. S. Amto , L. M. Weinstock , Tetrahedron Lett. 1984, 25, 4337–4340;

[14b] A. L. J. Beckwith , C. L. L. Chai , J. Chem. Soc. Chem. Commun. 1990, 1087–1088.

[15] For recent synthetic approaches exploiting diastereoselective radical additions to the Karady-Beckwith alkene, see:

[15a] R. A. Aycock , C. J. Pratt , N. T. Jui , ACS Catal. 2018, 8, 9115–9119;

[15b] A. Trowbridge , D. Reich , M. J. Gaunt , Nature 2018, 561, 522–527; 30258135 10.1038/s41586-018-0537-9

[15c] P. Ji , Y. Zhang , Y. Dong , H. Huang , Y. Wei , W. Wang , Org. Lett. 2020, 22, 1557–1562; 32045253 10.1021/acs.orglett.0c00154 PMC7936574

[15d] K. Merkens , F. J. Aguilar Troyano , J. Djossou , A. Gómez-Suárez , Adv. Synth. Catal. 2020, 362, 2354–2359;

[15e] O. Zhang , J. W. Schubert , J. Org. Chem. 2020, 85, 6225–6232. 32268730 10.1021/acs.joc.0c00635

[16] D. E. Palmer , C. Pattaroni , K. Nunami , R. K. Chadha , M. Goodman , T. Wakamiya , K. Fukase , S. Horimoto , M. Kitazawa , J. Am. Chem. Soc. 1992, 114, 5634–5642.

[17] In nature—for example, in flavin binding LOV domains—similar radical pairs undergo coupling. This likely does not occur here as the radicals are not caged by the protein matrix. J. Herrou , S. Crosson , Nat. Rev. Microbiol. 2011, 9, 713–723. 21822294 10.1038/nrmicro2622 PMC3286519

